# Wholesome Heat for Healthful Homes

**Published:** 1872-04

**Authors:** Ben. H. Pratt


### THE BISTOURY.

VOL. VIII. ELMIRA, N. Y., APRIL, 1872. NO. 1.

### Communications.

### WHOLESOME HEAT FOR HEALTHFUL HOMES.

BEN. H. PRATT, A. M.

There is much talk about it.

There is large reason why there should be much talk about it.

Not that there isn't a great deal of nonsense and stuff and chaff in all the pro-ing and con-ing upon this important heat question—house heat—home heat; and a multitude of opinions thereupon, as to the apparatus necessary to make it—the quantity desired—the quality needed.

Upon what subject is there not ultra talk and windy gabble now a-days?

Theories? no end of them! Theorists? aye, as the sands of the sea for multitude! and variable as the mind of womankind.

Smith advocates Grates—low down or otherwise. Brown has things he calls Baltimore Heaters, and likes them. Jones has stoves all over his house. White has a miniature forge down in the lower regions somewhere, and imagines himself a happy Vulcan. Green isn't a timid man, so he charges his home with caloric begotten of a boiler, from which steam makes vermicular twists through every room of his dwelling.

Smith, Brown, Jones, White and Green all get heat. I shan't deny that they all get enough. I might, but I wont. Say they all get the desired quantity, as we must admit *something*.

Smith, the Grate man, gets
- Heat (most of which goes up chimney.)
- Air (plenty of that—but he likes it.)
- Dust (orthodox, for "from dust thou art.")
- Labor (of the hod-carrying sort.)
- Cheer (enough to balance dust and labor?)

Brown, the Baltimore H. man, gets
- Heat, (one-half his—t'other chimney's.)
- Bad air, (oxygen all burnt out of it.)
- Dust and Labor, (Smith sympathises.)
- Cheer, (none to brag of.)
- Gas, (but not economised.)

Jones, the stove fancier, gets,
- Heat, (in spots and uncertain.)
- Bad Air, (in same boat with Brown.)
- Dust, (worse and more of it, but not fatal.)
- Labor, ( " " " " " very nearly ")
- Cheer? (goes to his neighbors for it.)
- Gas, (See Brown and double the dose.)
- Wear and Tear, (house and furniture.)
- Agony, (mental and physical.)
- Profanity, (annual, autumnal spells, when piping.)

White, the Vulcan, gets
- Heat, (even and sufficient.)
- Air, (pure and plenty.)
- Dust, (not a particle.)
- Labor, (arduous, but not frequent.)
- Cheer, (not directly.)
- Gas, (none, if careful.)
- Wear and Tear, (none.)

Green, the Steamer, gets
- Heat, (delightful, but scant.)
- Air, (not burned, but close.)
- Dust, (none.)
- Labor, (little.)
- Crackling noise, (expansion and contraction.)
- Cheer, (yielded for comfort.)
- Gas, (unknown.)
- Wear and Tear, (out of account.)

One might infer that economy—cost of apparatus and fuel—were a constant quantity in each method, but popular opinion makes the stove user the saver, and the advocate of steam the most extravagant, the scale running like this, calling No. 1 the most economical:

No. 1—Stove,
" 2—Furnace,
" 3—Baltimore Heater,
" 4—Grate,
" 5—Steam.

The Formulæ, then, which we might suppose Smith, Brown, Jones, White and Green using to represent their varied adoptions, would appear somewhat like this, the left of the equation representing the gains, and the right the losses:

Smith. Air+Cheer+Room=Heat less 60 pr. ct.+Dust+Labor+Burnt face+cold back.

Brown. Room=Heat less 50 pr. ct.+Bad air +Dust+Labor+Cheer+Gas.

Jones. Heat = Bad air + Dust + Labor + Cheer+Gas+Wear+Tear+Agony+Profanity.

White. Heat+Air=Labor.

Green. Heat+Air=Labor+Noise.

From an examination of the above equations we must draw a conclusion. To our mind it lies between White's and Green's choice, with a decided leaning toward White, since his is free from noise and his labor is but little greater than Green's.

And yet what a world of prejudice there is against Heaters in general. One always imagines a severe pain in the head upon entering a dwelling heated in this way. Another wants to *see* the heating medium, and can't be warm unless he does. As if heat were communicated to the body through the eyes. Another delights in the glow of the coals and grows eloqent, if not sentimental, over the pleasure derived from reveries over burning coals, but takes no thought of the chills that chase themselves up and down the back meanwhile. But the newest nonsense is developed by the advocates of the concerns known as the Baltimore Heaters, who have the mania once, like the measels, only it lasts one and sometimes two years, when the patient becomes rational again and a caloric revolution takes place in the household. The Steam Heating apparatus is so peculiar in its construction, and so expensive in its introduction, and withal so nearly akin to the Furnace in its general results, that they who have adopted it seldom abandon it, though tacitly admitting its defects. To me, the principle upon which the basement heater works, seems the true one. The great lack in every other method is the absence of pure air, except that which is admitted from out of doors by means of doors, windows or other openings. This is effectual, if resorted to, but the change is so sudden, so violent, that colds or discomfort are sure to arise from it. In the basement furnace you have a cold air pipe, communicating with the outer air, through which is carried pure air into your furnace, and thence, tempered to a degree of comfort, but not burned, carried directly to your apartments, taking the place of the air previously occupying them, and forcing out the older and used-up portion. In every house there are chinks and apertures for this purpose, unless my experience is faulty, and art cannot avoid them if it would. Again, the air forced through the hot air pipes causes a constant pressure of the atmosphere from within, outward, preventing in a great degree that influx of cold with which we are all so well acquainted, and to which almost every one is extremely sensitive. The simple fact that one is not compelled to breathe the same air over and over again, ought to claim from every seeker for and possessor of health a favorable opinion, even though the objections to furnace heat were fourfold what can be trumped up against it.

From what an annoyance, too, are we exempt, in the form of coal carrying and coal spilling and coal dust and ash ditto, ditto, ditto, and tracking through our rooms and over our carpets, soiling and making work, and bringing broom, dust-pan and duster into hourly requisition.

Oh! but some object on the score of economy. Well, I don't mean to argue the point, but it seems to me that a comparative estimate, made from a number of cases, would result somewhat like this: Say three stoves is the average number used in our dwellings, and that they each demand from one to three large scuttles of coal per day—a very moderate estimate, take the season through—and we have an average of two, and a total of six scuttles per day, and a house but imperfectly warmed. Now I have never used less than one nor more than six scuttles in my own furnace, upon any day, and am positive I could not use more than four on an average the season through, heating five rooms and a hall the whole time and sometimes an additional room. These rooms are large and high, and are beyond the average cubic dimensions of ordinary apartments. I do not offer this comparison as an accurate statement, but one drawn from casual observation of stove uses, and in the way the facts strike me. If I am correct, there is a large margin to the furnace user, more than sufficient to balance the difference in the cost of heating apparatus.

Moreover, the quality of the air, aside from its purity, coming through a well cared for furnace, becomes moist enough for grateful breathing, by reason of the water tanks over which it passes, on its way through, and does not reach you in the form of steam, as is necessary where water is kept on the top of the stove, as is the usual way.

Looking at the question of wholesome heat from every stand-point, I am at a loss to know how any thoughtful person can adopt other than this method of securing it. And yet there be those in whose judgment I have great confidence, decrying the use of the furnace and advocating the use of the stove. I can but think that the prejudices of such have been brought about by an imperfect or ill-constructed heater —and there are many such—and only need the convincing proof which a well constructed and well managed furnace will afford them. To these I would earnestly advise an early investigation of the subject, confident in the belief that an insight into the real merits of this comfort, will lead to its adoption by many who are now only enduring their present method until they can find, or some one can invent a better. I do not believe that house owners, ten years hence, will admit a heating stove into their dwellings, and the renters alone will be the unwilling users of that which is only one remove better than no heater, the stove.

